# Dysfunction of alveolar macrophages after cardiac surgery and postoperative pneumonia? - an observational study

**DOI:** 10.1186/cc13148

**Published:** 2013-12-09

**Authors:** Katharina Chalk, Christian Meisel, Claudia Spies, Thomas Volk, Karin Thuenemann, Jörg Linneweber, Klaus-Dieter Wernecke, Michael Sander

**Affiliations:** 1 Department of Anesthesiology and Intensive Care Medicine, Campus Charité Mitte and Campus Virchow-Klinikum, Charité - Universitätsmedizin Berlin, Charitéplatz 1, 10117, Berlin, Germany; 2 Department of Immunology, Charité-Universitätsmedizin Berlin, Campus Virchow-Klinikum, Berlin, Germany; 3 Department of Anesthesiology, Intensive Care and Pain Therapy, Saarland University Hospital, Homburg, Germany; 4 Department of Cardiovascular Surgery, Charité-Universitätsmedizin Berlin, Campus Charité Mitte, Berlin, Germany; 5 Sostana GmbH, Wildensteiner Strasse 27, 10318 Berlin, Germany

## Abstract

**Introduction:**

Patients undergoing cardiac surgery have an increased risk of postoperative pneumonia. Pulmonary immune dysfunction might be a contributing factor. We therefore determined changes of the surface molecules on alveolar macrophages (AMs). To characterize modulation in patients with pneumonia we correlated these changes to the development of postoperative pneumonia.

**Methods:**

After ethical approval and written informed consent, 33 patients undergoing elective coronary bypass grafting surgery were included in this observational study. Peripheral blood cells and alveolar lavage fluid were collected directly after induction of anesthesia and two hours after separation from cardiopulmonary bypass (CPB). Human leukocyte antigen-DR (HLA-DR) and toll-like receptors (TLR) 2/4 expression on monocytes and AM were assessed by flow cytometry. A total of three patients developed postoperative pneumonia determined according to the criteria of the Center of Disease Control. Statistical analysis was performed with the Mann–Whitney-*U* test and Wilcoxon test.

**Results:**

We found significant changes of phenotypic and functional immune markers on AMs after cardiac surgery. HLA-DR expression on peripheral blood monocytes and AMs was significantly reduced compared to baseline in all patients (each approximately 30%). After surgery patients who developed postoperative pneumonia revealed a trend of stronger reduction of HLA-DR expression (83.7% versus 27.1%) and TLR4 expression on AMs (46.1% versus 9.9%) compared to patients without pneumonia. Already before surgery, the baseline of TLR2 expression on AM was significantly lower (27.7%) in patients who developed postoperative pneumonia.

**Conclusions:**

As far as we know this is the first study that shows an early impairment of lung cellular immune response after cardiac surgery. These findings can help to understand the role of cell-mediated immunosuppression and its association to the development of postoperative pneumonia.

## Introduction

Patients undergoing cardiac surgery with cardiopulmonary bypass (CPB) have a high risk for nosocomial infections [1]. The most common infection is pneumonia (approximately 5 to 8%), causing a longer hospital stay and higher mortality [2-4]. In particular, gram-negative bacteria, such as *Klebsiella pneumoniae,* are common pathogens after cardiac surgery [4,5]. Perioperative dysregulation of the pulmonary defense system may be associated with this frequent complication [6-8].

Alveolar macrophages (AMs) are one of the major resident effector-cells of the pulmonary defense system against respiratory tract pathogens. These local antigen-presenting cells express human leukocyte antigen-DR (HLA-DR) protein, part of the major histocompatability complex (MHC) class II system, that activates T lymphocytes and B cells [9]. It has been shown that patients with decreased HLA-DR levels (mHLA-DR) on circulating peripheral blood monocytes have a higher risk for infections after surgery [10-12]. Therefore, mHLA-DR expression has been proposed as a tool to predict the risk of secondary infection [9]. Lukaszewicz *et al*. reported in a large study of 283 ICU patients an association between persistent low mHLA-DR expression and the development of nosocomial infections [12]. Several immune-stimulating treatments (interferon γ and granulocyte-macrophage colony-stimulating factor) in septic patients could improve immune reactivity as indicated by increasing mHLA-DR levels and monocytic cytokine secretion [13,14]. A prospective clinical trial in 40 patients with septic neutropenic acute respiratory distress syndrome (ARDS) suggests that decreased levels of HLA-DR on AMs in the lung predispose to the development of pulmonary infections [15]. These findings are supported by a study of Muehlsted *et al*. who found a correlation between the incidence of nosocomial pneumonia and a prolonged reduction of HLA-DR expression on AMs in injured patients [16].

Pathogens can be sensed by AM via toll-like receptors (TLRs). Lipopolysaccharide (LPS), the major component of gram-negative bacterial cell walls, activate AM through the TLR4 receptor as a part of the LPS receptor complex [17]. Other diverse bacterial elements, including peptidoglycans from gram-positive bacteria, bind to TLR2 [18]. Several experimental studies demonstrated that TLR4 and TLR2 are crucial for the defense against nosocomial pathogens including *K. pneumoniae*[19]. The binding of bacterial cell wall components to TLRs on AMs results in the release of cytokines and chemokines which induce the chemotaxis and activation of T cells and neutrophils [20,21]. Hadley *et al*. showed a reduction of 29% of the TLR expression on monocytes at the end of CPB. His workgroup was unable to link the reduced cytokine production to the downregulation of TLR expression but assumed that upregulation could contribute to the recovery of the immune responsiveness [22].

Even though the systemic inflammatory response to cardiac surgery with CPB is well-characterized [23-26], the understanding of the local pulmonary effects remains incomplete. We hypothesized that there is a suppression of HLA-DR expression on AMs in patients developing postoperative pneumonia compared to patients without this complication. Therefore, we tried to characterize the effect of cardiac surgery with CPB on early functional changes of AMs in this pilot observatory study and correlated these findings to the development of postoperative pneumonia.

## Material and methods

### Patients

After approval from the local ethics committee - the Ethics Committee of the Charité - Universitätsmedizin Berlin (EA1/192/05) - and informed written consent from 33 patients undergoing elective coronary artery bypass grafting (CABG), patients were enrolled in this prospective clinical pilot trial. Inclusion criteria were age >18 years and undergoing elective CABG surgery. Exclusion criteria were missing signed informed consent, age <18 years, pregnancy, lung disease with ambulatory oxygen respirator, liver insufficiency (Child-Pugh classification > B), HIV infection, corticosteroid therapy, status post organ transplantation and preoperative signs of infection according to the criteria of the Center of Disease Control, Germany. Postoperative pneumonia was diagnosed with the criteria of the Center of Disease Control.

### Anesthetic, surgical course and intensive care management

Patients were given etomidate, fentanyl and cis-atracurium for induction and sevoflurane and fentanyl for maintenance of the general anesthesia. An arterial catheter via the left radial artery was used for measurement of arterial blood pressure and to obtain blood samples for blood gas analysis.

The standardized prime for the CBP circuit consisted of 500 ml of crystalloid fluid and 500 ml of 10% hydroxyethylstarch solution. A total dose of 50,000 KIU aprotinin per kg bodyweight, 8,000 IE heparin, 250 ml mannitol (20%) and 1 g methylprednisolone were administered during CPB in all patients. All patients were monitored for at least 24 hours at the ICU.

### Sample extraction

#### Blood sampling

Arterial blood gas analyzes (BGA) and peripheral blood samples (ethylenediaminetetraacetic acid (EDTA), heparin, vacutainer, Becton Dickenson (BD), Heidelberg, Germany) were taken before and after bronchoscopy prior to and after surgery.

#### Bronchoalveolar lavage

After induction of anesthesia before surgery, and two hours after separation from CPB we collected the bronchiolar lavage (BAL) fluid. Patients were ventilated with a fraction of inspired oxygen of 1.0 during the intervention. After placement of the bronchoscope in the wedge position, 100 ml of saline solution (0.9%) was used preoperatively to aspirate cells from the right middle lobe and postoperatively from the left lingula of the lung. The aspirated fluid was filtered (cell strainer 100 μm, BD), centrifuged (10 minutes, 300 J) and the pellet resuspended in culture medium (Roswell Park Memorial Institute medium (RPMI), PAA Laboratory GmBH, Pasching, Austria). The cells were counted in the counting chamber (Neubauer, LO Laboroptic, Friedrichsdorfs, Germany) and adjusted to a concentration of 5 × 10^6^ cells per ml.

### Differential cell counts

For flow cytometry (FACS) analysis the following fluorescence-labeled mouse anti-human monoclonal antibodies (BD Biosciences; San Diego, CA, USA) were used: cluster of differentiation (CD) 45 peridinin chlorophyll; CD2, CD16 fluorescein isothiocyanate (FITC); CD3 and CD19 phycoerythrin (PE); CD14 allophycocyanine (APC). Briefly, 50 μl of EDTA blood or BAL cell suspension was stained with fluorescently-labeled antibodies 30 minutes in the dark at 4°C, washed and resuspended in PBS with 2% FCS. Samples were stored on ice until FACS analysis. Cell phenotyping was performed by four-color flow cytometry on a FACSCalibur™ using CELLQuest™ software (BD Biosciences).

### Surface markers on mononuclear cells

#### HLA-DR expression

For quantitative measurement of HLA-DR expression on monocytes and AMs, 50 μl of blood or BAL cell suspension, respectively, was incubated with 20 μl QuantiBrite Anti-HLA-DR PE/Anti-Monocyte PerCP-Cy5.5 (BD) reagent for 30 minutes at room temperature. Blood samples were additionally incubated with 500 μl FACS lysing solution (BD) for 15 minutes at room temperature for red blood cell lysis. Cells were kept 4°C after washing, until analysis by flow cytometry.

#### TLR2/4 expression

The measurement of TLR2/4 expression was performed using two different AB mixes: For analysis of TLR2 expression a mix of CD45 peridinin chlorophyll, CD 14-APC, (BD), TLR2-FITC and IgG2a-PE was used (BD Biosciences). The TLR4 expression was measured with CD45 peridinin chlorophyll, CD 14-APC, (BD), TLR4-PE and IgG2a-FITC (BD Biosciences). Each mixture was incubated with BAL cells (50 μl cells) for 30 minutes at 4°C and after one washing step was resuspended in buffer solution (BD). Cells were stored on ice until the flow cytometric measurements were performed.

### Statistical methods

Owing to the limited sample sizes and asymmetrically distributed observations we used nonparametric statistical tests. Results are expressed as median and IQR for continuous variables and percentage of frequencies for categorical data: 95% confidence intervals were calculated. The data were analyzed with the non-parametric Wilcoxon test for pairwise comparisons and the non-parametric Mann Whitney *U*-test for independent groups of patients. To calculate the relative changes (%) between the groups the median values were used. *P* <0.05 was considered statistically significant. Statistical analysis was carried out using the Software Package for Social Sciences, 18.0 SPSS® for Windows® (SPSS, Inc., Chicago, IL, USA).

## Results

### Patients’ characteristics

Peripheral blood and BAL samples were obtained from all 33 patients. The baseline characteristics are given in Table 1. Retrospectively the patients were divided into group 1 (those without postoperative pneumonia, n = 30) and group 2 (those with postoperative pneumonia, n = 3).

**Table 1:** Baseline characteristics of the analyzed patient groups

**Characteristics**	**Group 1 without pneumonia (n = 30)**	**Group 2 with pneumonia (n = 3)**	***P***
Age, years	67 (59, 72)	79 (78, 84)	0.006^a^
Gender, m/f	22 (73%)/8 (27%)	2 (66%)/1 (33%)	0.88
Smoker, %	3 (10%)	0 (0%)	0.49
BMI, kg m^-2^	27.5 (25.5, 29.7)	26.9 (22.5, 28.03)	0.45
LVEF, %	55 (50, 60)	55 (50, 66)	0.75
Arterial hypertension, n/y	2/31	0/3	0.88
COPD, n/y	31/2	3/0	0.88
Renal insufficiency, %	2 (6.7%)	1 (33%)	0.49
Diabetes mellitus, %	6 (20%)	1 (33%)	0.75
Duration of anesthesia, minutes	270 (234, 318)	360 (330, 360)	0.019^a^
Duration of surgery, minutes	213 (173, 244)	275 (275, 320)	0.025^a^
CPB-time, minutes	73 (54, 99)	125 (101, 142)	0.030 ^a^
Cross clamp time, minutes	41 (32, 61)	58 (40, 97)	0.235
Total ventilation time, hours	13 (11.0, 15)	24 (22, 25)	0.015^a^
Intraoperative blood transfusion, n/y	7 (23%)	3 (100%)	0.025^a^
Postoperativewound infection, %	4 (13.3%)	0 (0%)	0.75
Onset of pneumonia, postoperative day	n/a	5 (4, 5)	0.10
Sepsis, %	0 (0%)	2 (67%)	0.06

Data in brackets are presented as median and IQR or absolute values. ^a^Statistically significant (*P* <0.05) (Mann–Whitney *U*-test for independent patient groups). Patients were divided retrospectively into two groups: group 1 without postoperative pneumonia and group 2 comprising three patients who developed postoperative pneumonia. m/f, male/female; n/y, no/yes; COPD, chronic obstructive pulmonary disease; CPB, cardiopulmonary bypass; n/a, not applicable.

The patients in group 2 all had arterial hypertension and one patient had renal insufficiency and diabetes mellitus. They were all non-smokers and had no chronic obstructive pulmonary disease (COPD). All patients with postoperative pneumonia had prolonged CPB and ventilation times and received intraoperative blood transfusions. Two patients with postoperative pneumonia developed severe sepsis, died within 60 days. The demographic data were similar in all patients. In group 2 the patients were significantly older and had significantly longer duration of surgery and CPB. Relevant pre-existing medical conditions were mainly arterial hypertension, and in a small patient group there was also COPD, renal insufficiency and diabetes mellitus.

Ten patients received blood products during surgery. Seven patients developed postoperative infection: four developed wound infection and three developed postoperative pneumonia. The incidence of postoperative pneumonia was 9%. The median of the onset of pneumonia was on postoperative day (POD) 5. Two of the patients with postoperative pneumonia developed septic shock and lethal severe sepsis whilst in the ICU.

### Cell counts and differential staining

The systemic leukocyte counts were within normal range before surgery: 7.3/μL (6.2/μL to 8.8/μL) and were significantly elevated after surgery 10.3/μL (9.2/μL to 13.8/μL) (*P* = 0.001) and on the first POD 12.8/μL (11.5/μL to 14.2/μL) (*P* = 0.001) compared to preoperative values. The peripheral blood samples showed significant changes in the differential subsets with a reduced percentage of monocytes and lymphocytes after surgery for all patients. Neutrophil counts were significantly higher compared to preoperative samples for all patients (data given in Table 2).

**Table 2:** Differential subsets of peripheral blood and bronchoalveolar lavage samples

	**Peripheral blood subsets (%)**			**Bronchoalveolar subsets (%)**		
**Time point**	**Preoperative**	**Postoperative**	***P***	**Preoperative**	**Postoperative**	***P***
**Macrophages (n = 33)**	n/a	n/a		63.5 (38.8, 76.5)	52.2 (21.5, 69.6)	0.018^a^
Group 1 (n = 30)	n/a	n/a		63.2 (35.2, 73.0)	53.4 (22.0, 70.2)	0.06
Group 2 (n = 3)	n/a	n/a		77.1 (56.7, 85.7)	27.9 (7.5, 57.4)	n/a
**Monocytes (n = 33)**	8.0 (6.7, 9.9)	4.0 (2.6, 5.7)	0.001^a^	3.4 (2.3, 4.6)	3.1 (1.8, 4.7)	0.83
Group 1 (n = 30)	7.9 (6.7, 10.2)	4.4 (2.8, 6.0)	0.001^a^	3.3 (2.3, 4.2)	3.2 (2.0, 4.7)	0.758
Group 2 (n = 3)	9.3 (7.1, 9.6)	2.3 (2.2, 4.0)	n/a	4.3 (2.4, 11.9)	1.8 (1.7, 7.5)	n/a
**Lymphocytes (n = 33)**	21.4 (17.1, 30.3)	7.4 (5.8, 11.9)	0.001^a^	11.4 (7.4, 14.5)	9.2 (4.6, 17.1)	0.99
Group 1 (n = 30)	21.6 (17.5, 29.7)	7.5 (5.7, 11.7)	0.001^a^	11.5 (7.7, 15.7)	9.9 (5.7, 19.2)	0.673
Group 2 (n = 3)	6.4 (5.8, 34.4)	6.9 (6.2, 10.2)	n/a	13.1 (4.7, 14.2)	4.3 (3.4, 11.4)	n/a
**Neutrophils (n = 33)**	66.7 (57.7, 74.3)	87.4 (81.4, 91.2)	0.001^a^	10.6 (3.1, 28.8)	15.1 (2.1, 46.7)	0.088
Group 1 (n = 30)	66.6 (57.8, 74.0)	87.2 (80.4, 91.3)	0.001^a^	11.3 (3.1, 35.5)	14.3 (1.8, 41.0)	0.254
Group 2 (n = 3)	82.4 (56.6, 83.5)	88.9 (87.4, 91.3)	n/a	4.8 (3.1, 11.7)	45.2 (15.1, 82.7)	n/a

In the peripheral blood samples (n = 33) the monocytes and lymphocytes were significantly reduced after surgery, whereas the neutrophils were significantly elevated. The findings in the bronchiolar lavage samples (n = 33) were different. The trend was similar to the blood samples but only the percentage of macrophages was significantly reduced after surgery. Data are given as medians and IQR in brackets. The Wilcoxon test was used to calculate significant difference for the dependent variables; ^a^significantly different; n/a, not available.

BAL samples showed similar differential subsets before and after surgery. The percentage of AMs after surgery was reduced by trend in group 1 (*P* = 0.06). In patients with postoperative pneumonia (group 2) AMs showed a strong reduction after surgery (*P* = n/a) (data given in Table 2).

### HLA-DR expression on peripheral blood monocytes and AMs

HLA-DR expression on peripheral blood monocytes (n = 31) was significantly diminished after surgery in the whole patient population (*P* = 0.001). In patient group 1 mHLA-DR expression went down significantly (*P* = 0.001). In group 2 mHLA-DR was also strongly reduced by trend (*P* = n/a). Comparing the two groups (1 vs. 2) mHLA-DR expressions did not differ before (*P* = 0.97) or after surgery (*P* = 0.12) (data given in Table 3).

**Table 3:** HLA-DR expression (antibody/cell) on peripheral blood monocytes and AMs

	**Peripheral blood monocytes**			**AMs**		
**Time point**	**Preoperative**	**Postoperative**	**p**	**Preoperative**	**Postoperative**	***P***
All (n = 31)	26,587 (20,410, 31,478)	13,996 (11,724, 17,706)	0.001^a^	985,234 (698,683, 1,293,531)	712,564 (320,726, 941,120)	0.001^a^
Group 1 (n = 28)	26,266 (20,646, 31,415)	15,258 (12,365, 18,580)	0.001^a^	1,009,337 (739,280, 1,294,545)	736,306 (430,604, 943,491)	0.002^a^
Group 2 (n = 3)	27,882 (12,325, 34,088)	10,292 (10,288, 13,389)	n/a	652,262 (505,628, 985,234)	106,139 (42,434, 417,111)	n/a

HLA-DR expression on peripheral blood monocytes as well as on AMs was significantly reduced after surgery. In group 2 a strong reduction without statistical significance was seen. Data are given as medians and IQR in brackets. The Wilcoxon test was used to calculate significant difference for the depending variables; ^a^statistically significant difference. HLA-DR, human leukocyte antigen-DR; AM, alveolar macrophage.

**Figure 1:**
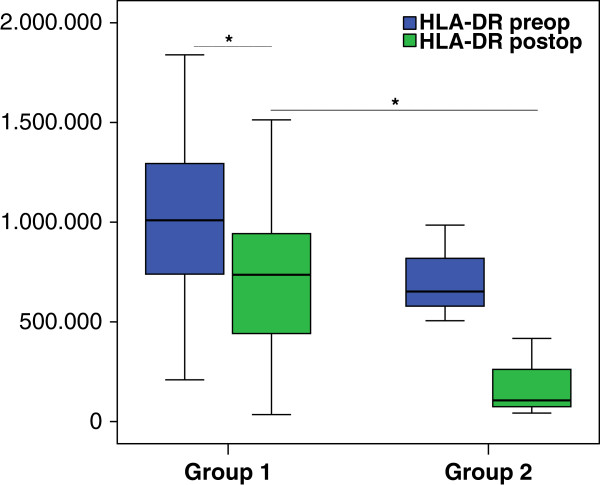
**Human leukocyte antigen-DR (HLA-DR) expression on alveolar macrophages (AMs).** HLA-DR expression on AM in group 1 (n = 28) showed a significant reduction after surgery (*P* = 0.002). In group 2 (n = 3), HLA-DR expression was highly diminished after surgery not possible to calculate *P* value with only 3 patients. Comparing both groups the baseline had a tendency to be lower (*P* = 0.20) and the postoperative HLA-DR expression was significantly reduced in group 2 (*P* = 0.024). Box plots represent median and IQR; blue, values before surgery; green, values after surgery. The Wilcoxon test was used for dependent groups and the Mann–Whitney *U*-test for independent groups; ^*^P <0.05.

HLA-DR expression on AMs (n = 31) was also significantly reduced after surgery in the whole patient population (*P* = 0.001). Group 1 was significantly diminished (*P* = 0.002) as well as a strong reduction in Group 2 (*P* not applicable (n/a)) (Figure 1).

Comparing the two groups (1 versus 2), in group 2 the baseline HLA-DR expression was lower by trend (*P* = 0.20) and the postoperative values were significantly reduced (*P* = 0.024).

### TLR2/4 expression on AMs

We found no significant changes of TLR2/4 expression on AMs after surgery in the whole patient population (n = 32): for TLR2 *P* = 0.31, and for TLR4 *P* = 0.77 (data given in Table 4).

**Table 4:** TLR2/4 expression (antibody/cell) on AMs

	**TLR2**			**TLR4**		
**Time point**	**Preoperative**	**Postoperative**	***P***	**Preoperative**	**Postoperative**	***P***
All (n = 32)	55.2 (46.0, 81.9)	52.1 (41.2, 72.0)	0.31	63.6 (48.4, 94.1)	57.0 (41.7, 87.4)	0.77
Group 1 (n = 29)	59.3 (51.0, 84.5)	52.2 (41.4, 76.1)	0.32	64.6 (48.9, 97.9)	58.2 (43.7, 89.8)	0.15
Group 2 (n = 3)	42.9 (34.6, 43.0)	30.0 (22.1, 58.1)	n/a	62.7 (36.4, 75.2)	33.8 (33.8, 36.1)	n/a

TLR2 and TLR4 levels did not differ before and after surgery. In group 2 the TLR4 levels were strongly reduced by trend after surgery. Data are given as medians and IQR in brackets. The Wilcoxon test was used to calculate significant difference for the dependent variables. AM, alveolar macrophage; TLR, toll-like receptor.

**Figure 2:**
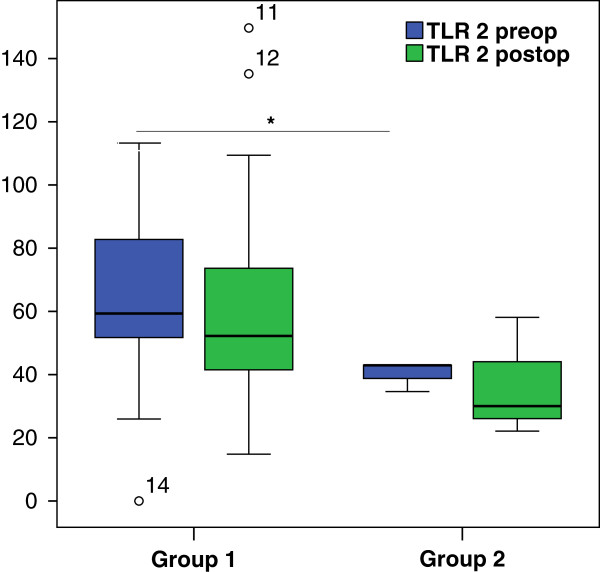
**Toll-like receptor (TLR)2 expression on alveolar macrophages (AMs).** TLR2 expression in group 1 (n = 29) did not differ before and after surgery (*P* = 0.32). In group 2 (n = 3) the TLR2 expression was lower by trend after surgery not possible to calculate *P* value with 3 patients (as in Figure 1 and 3). Comparing both groups, group 2 showed a significantly lower baseline of TLR2 expression (*P* = 0.027) and a tendency of lower postoperative values (*P* = 0.21). Box plots represent median and IQR; blue, values before surgery; green, values after surgery. The Wilcoxon test was used for dependent groups and the Mann–Whitney *U*-Test for independent groups; ^*^P <0.05.

**Figure 3:**
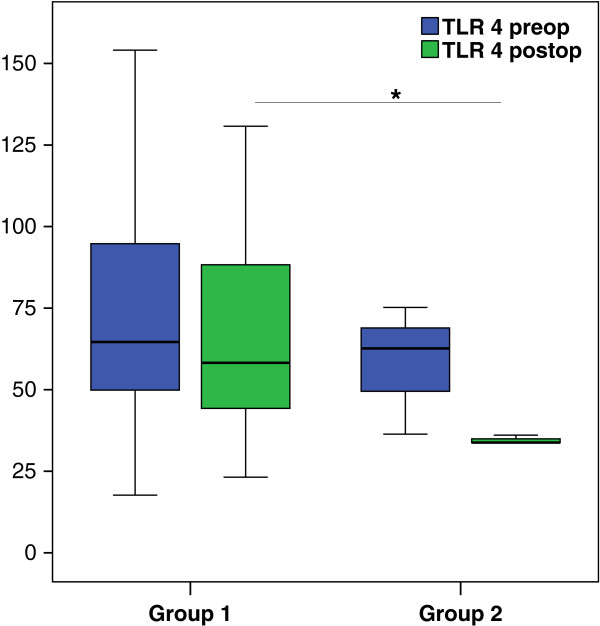
**Toll-like receptor (TLR)4 expression on alveolar macrophages (AMs).** The TLR4 expression did not differ before or after surgery in group 1 (n = 29) (*P* = 0.15). In group 2 (n = 3), TLR4 expression tended to be reduced (*P* not applicable (n/a)). Comparing the groups the baseline of TLR4 did not differ (*P* = 0.45). However, after surgery the TLR4 expression was significantly diminished in group 2 (*P* = 0.041). Box plots represent median and IQR; blue, values before surgery; green, values after surgery. The Wilcoxon test was used for dependent groups and the Mann–Whitney *U*-test for independent groups; ^*^P <0.05.

The TLR2 expression in group 1 (*P* = 0.32) and group 2 (*P* = n/a) was not different before and after surgery (Figure 2). TLR4 expression in group 1 showed similar results before surgery and after surgery (*P* = 0.15) (Figure 3). In group 2 the TLR4 the expression was reduced by trend after surgery (*P* = n/a) (Figure 3).

Interestingly, comparing the two groups (1 versus 2) we found a significantly lower baseline TLR2 (*P* = 0.027) in group 2, whereas the baseline TLR4 did not differ (*P* = 0.45). However, postoperatively in group 2 the TLR4 expression was significantly reduced (*P* = 0.041) additionally to the trend for lower TLR2 expression (*P* = 0.21).

## Discussion

Our results show significant changes in phenotypic and functional immune markers on AMs early after cardiac surgery. In addition to the well-known systemic immune impairment there seems to be a pulmonary immunosuppression characterized by a diminished HLA-DR expression on AM after surgery. The three patients with postoperative pneumonia (9%) were characterized preoperatively by a lower baseline HLA-DR (−35.4%) and TLR2 expression (−27.7%). After surgery the expression of HLA-DR (−83.7%), as well as the TLR4 expression (−46.1%), was significantly reduced.

Cardiac surgery poses a high risk for the development of postoperative pneumonia, causing high morbidity and mortality in these patients [1-4]. The early identification of patients at risk for the development of postoperative pneumonia would be desirable, however is still difficult. In recent years various attempts have been made to find markers and risk factors to predict the development of nosocomial pneumonia after cardiac surgery. A large cohort study in the USA in 17,145 patients undergoing cardiac surgery was performed to clarify the derivation and validation of postoperative pneumonia [27]. In this study only 361 patients (2%) developed pneumonia. They were able to identify thirteen independent predictors for postoperative pneumonia, for example body mass index <18.5, smoking history, creatinine level over 1.2 mg/dl, blood transfusion, mechanical ventilation time >24 hours, cancer history and emergency status. The authors concluded that it may be useful to prove the concept of preventive interventions [27]. However, specificity of this risk model seems to be poor and prevents implementation of preventive strategies. Another cohort study from Brazil in 331 patients with a rate of 16% for postoperative pneumonia comes to the conclusion that preoperative level of highly sensitive C-reactive protein (CRP) >3 mg/l is an independent predictor for postoperative respiratory infection [28]. However, specificity is also problematic with this approach, as CRP is not only increased in pulmonary infections, but also in a variety of comorbidities. The key to understanding postoperative pulmonary infection might be the local host-response to pathogens and the resulting pattern of immune markers. This might help to obtain insights into the immune response after surgery and the associated change with localized infections.

Muehlstedt *et al*. showed a correlation between low HLA-DR levels on AMs and pneumonia in injured patients. The authors demonstrated that the reduced HLA-DR expression on AMs preceded nosocomial pneumonia and they suggested that a local immune suppression of the lung with altered effector cell function could be responsible [16]. In their setting they compared patients 12 hours after injury, to a control group of healthy volunteers. Six of sixteen patients developed nosocomial pneumonia and had persistent low HLA-DR expression on AMs, whereas in the patients without pneumonia the HLA-DR expression returned to normal 60 hours after injury [16]. In our patient group the population of AMs was reduced 2 hours after separation from CBP with a significantly lower ability to express HLA-DR in patients who developed postoperative pneumonia. These data suggest that AM function is compromised already at this early time point. Unfortunately we were not able to examine the pulmonary immune function at a later time point as it was unethical to prolong the ventilation time longer than necessary. Since the onset of pneumonia was on day five, it might be possible that the function of AMs remains reduced for several days. This possible explanation needs to be addressed in further investigations.

In agreement with other studies our data show that patients after cardiac surgery with CPB have strong systemic immune depression [23,24,26]. The peripheral blood subsets were altered with significant reduction of monocytes and lymphocytes, and significantly higher neutrophil populations after surgery. In addition, mHLA-DR expression was significantly reduced after surgery. Large clinical trials have already suggested that persisting low levels of mHLA-DR expression could be used as an useful biomarker for the development of nosocomial infection in ICU patients [9,12].

Assessment of monocytic HLA-DR expression as an indicator for patients at risk for postoperative pneumonia is relatively easy to implement in clinical routine diagnostics. In this study, we also observed reduced HLA-DR levels on monocytes at 2 hours after surgery with a reduction of median values of 33.6% compared to baseline. However, our results failed to show significant differences between patients who did or did not develop pneumonia at this early point, likely due to the small sample size. Therefore, the hypothesis that the early local immunosuppression in the lung could be associated with higher susceptibility to developing postoperative pneumonia needs further investigation.

Additionally, in patients who developed postoperative pneumonia, we found significantly lower TLR2 levels on AMs even before surgery. One possible hypothesis is that this might be a sign of prevalence of bacterial colonization of the respiratory tract. In the current literature we found no data about the prevalence of airway bacterial colonization in healthy patients, but in stable COPD patients a colonization rate of over 50% has been seen [29,30]. Possibly, the strong surgical stress with mechanical ventilation permits pathogens colonizing the respiratory tract to proliferate. Unfortunately we did not take microbiological samples in this study. However, we are planning to investigate this aspect in further studies.

After surgery the TLR4 levels were significantly reduced on AMs. These findings might contribute to development of postoperative pneumonia, as other trials have been able to link the importance of TLR4 expression to the risk of pneumonia [19,31]. A small clinical study in six patients undergoing cardiac surgery described a reduction of systemic TLR2/4 expression on monocytes after surgery down to 29% that recovered up to 120% on the first postoperative day, indicating that receptor upregulation is a sign of recovery of responsiveness [22]. Unfortunately the incidence of postoperative infection was not part of this study. In contrast to the aforementioned study in our study design only early changes were analyzed. A recovery of the TLR4 expression on AMs on the first postoperative day might also be possible in our patient population.

Obviously, it would be helpful to compare our results for HLA-DR and TLR levels to healthy controls. However, in the literature to our best knowledge no such data have so far been published.

### Limitations

The major limitation of this study is the small group of patients who developed postoperative pneumonia. As this study was designed as a pilot study we were unable to include more patients at the time, however, we are planning a large interventional trial to verify our important findings. We cannot exclude activation of the AMs by the initial BAL in the right lobe before surgery. However, we tried to minimize these effects by performing the postoperative BAL in the left lobe. Furthermore, there might have been activation of the cells due to the laboratory protocol. However, as this was a clinical study in patients where all samples were treated equally, these manipulations could be disregarded as a confounding factor to our reported results. A multiplicity of factors such as age, gender, ventilation time, application of methylprednisolone and the use of blood products influence the perioperative immune function. Patients in group 2 were significantly older and had longer duration of surgery. These factors could also influence the levels of HLA-DR and TLR expression. Unfortunately, this pilot study has not the statistical power to identify or validate risk factors.

## Conclusions

Our results indicate that besides the systemic immune impairment after surgery an even stronger local immunosuppression in the lung might be a risk factor for pulmonary infections after cardiac surgery. Patients who developed pneumonia in this study showed a significant postoperative reduction of HLA-DR expression on AMs. This might indicate that concomitant to systemic immune dysfunction there is local dysregulation of the pulmonary compartment. These findings support the hypothesis of local cell-mediated immunosuppression and its association with the development of postoperative pneumonia. Further research needs to be performed to confirm the results from our group and others, and to put this into context of preventive measures.

## Key messages

• Alveolar macrophages are reduced after cardiac surgery

• Systemic and localized cell-mediated immune function is impaired after cardiac surgery

• Pronounced pulmonary cell-mediated immmunosuppression after cardiac surgery precedes pneumonia.

## Abbreviations

AB: Antibody; AM: Alveolar macrophage; APC: Allophycocyanine; BAL: Bronchoalveolar lavage; CABG: Coronary artery bypass grafting; CD: Cluster of differentiation; COPD: Chronic obstructive pulmonary disease; CPB: Cardio pulmonary bypass; CRP: C-reactive protein; EDTA: Ethylenediaminetetraacetic acid; FACS: Flow cytometry; FCS: Fetal calf serum; FITC: Fluorescein isothiocyanate; HLA-DR: Human leukocyte antigen-DR; LPS: Lipopolysacharide; n/a: not applicable; PBS: Phosphate-buffered saline; PE: Phycoerythrin; POD: Postoperative day; TLR: Toll-like receptor.

## Competing interests

The authors assure that there are no financial or non-financial competing interests.

## Authors’ contributions

MS, CM, TV and CS designed the study. KC, KT, MS, TV and JL enrolled subjects, gathered data and performed data management. The bronchoscopy was performed by MS and KC and blood samples were taken by MS, KC, JL and KT. Laboratory analysis was carried out by KC, CM, JL, TV and KT. KW, MS, TV, CS and KC performed the statistical analysis. Data interpretation was done by CS, MS, CM, KC, TV and KW. KC and CM wrote the manuscript. All authors read and approved the final manuscript.
